# Exploratory field study on the effects of porcine circovirus 2 (PCV-2) sow vaccination at different physiological stages mimicking blanket vaccination

**DOI:** 10.1186/s40813-021-00213-2

**Published:** 2021-04-26

**Authors:** Patricia Pleguezuelos, Marina Sibila, Raúl Cuadrado, Rosa López-Jiménez, Diego Pérez, Eva Huerta, Anna M. Llorens, José Ignacio Núñez, Joaquim Segalés, Sergio López-Soria

**Affiliations:** 1 IRTA, Centre de Recerca en Sanitat Animal (CReSA, IRTA-UAB), Campus de la Universitat Autònoma de Barcelona, 08193 Bellaterra, Spain; 2 OIE Collaborating Centre for the Research and Control of Emerging and Re-emerging Swine Diseases in Europe (IRTA-CReSA), Bellaterra, Spain; 3 UAB, Centre de Recerca en Sanitat Animal (CReSA, IRTA- UAB), Campus de la Universitat Autònoma de Barcelona, 08193 Bellaterra, Spain; 4 Departament de Sanitat i Anatomia Animals, Facultat de Veterinària, UAB, 08193 Bellaterra, Spain

**Keywords:** Porcine circovirus type 2, Blanket vaccination, Sow, Piglet, Production parameters

## Abstract

**Background:**

The objective of the present study was to explore the benefits of *Porcine circovirus 2* (PCV-2) blanket vaccination in a sow herd on productive parameters, PCV-2 infection and immune status in sows and their progeny. For this purpose, 288 sows were distributed among four balanced experimental groups. One group remained as negative control group and the other three received 1 mL of PCV-2 Ingelvac Circoflex® intramuscularly at different productive cycle moments: before mating, mid gestation (42–49 days post-insemination) or late gestation (86–93 days post-insemination); phosphate buffered saline (PBS) was used as negative control item. Reproductive parameters from sows during gestation and body weight of their progeny from birth to weaning were recorded. Additionally, blood was collected from sows at each vaccination time and piglets at 3 weeks of age. Moreover, up to 4 placental umbilical cords (PUC) per sow were taken at peri-partum. Sera from sows and piglets were analysed for PCV-2 antibody detection using an enzyme-linked immunosorbent assay (ELISA). Sera from sows and PUC were tested to quantify viraemia using a real time quantitative polymerase chain reaction (qPCR) assay.

**Results:**

Globally, results indicated that vaccinated sows showed heavier piglets at birth and at weaning, less cross-fostered piglets, lower viral load at farrowing as well as in PUC, and higher antibody levels at farrowing, compared to non-vaccinated ones. When all groups were compared among them, sows vaccinated at mid or late gestation had heavier piglets at birth than non-vaccinated sows, and lower proportion of PCV-2 positive PUC. Also, cross-fostering was less frequently practiced in sows vaccinated at pre-mating or mid gestation compared to non-vaccinated ones.

**Conclusions:**

In conclusion, the present study points out that PCV-2 sow vaccination at different time points of their physiological status (mimicking blanket vaccination) offers benefits at production and serological and virological levels.

**Supplementary Information:**

The online version contains supplementary material available at 10.1186/s40813-021-00213-2.

## Background

*Porcine circovirus 2* (PCV-2) is a small, circular, non-enveloped, single-stranded DNA virus of 20 nm in diameter approximately, belonging to the genus *Circovirus*, family *Circoviridae* [1]. This virus is ubiquitous in the pig population [2–6] and is the etiologic agent of clinical diseases and subclinical infection comprised under the term of porcine circovirus diseases [7].

Intrauterine PCV-2 infections in different stages of pregnancy may cause different reproductive disorders depending on the foetal immunological competence [8], the so-called PCV-2 reproductive disease. Intrauterine PCV-2 infection of foetuses, via insemination or transplacental, may cause late-term abortions, mummified, stillborn and weak born piglets [9–14]. Furthermore, pigs may be born PCV-2 viraemic due to transplacental infection that may subsequently act as an infection focus for their pen mates. All these situations imply that infected sows have a very important role in PCV-2 infection maintenance and dissemination in the herd due to horizontal and vertical infection transmission.

Nowadays, vaccination is a very effective tool to control PCV-2 infection. From 2007 to present, four major PCV-2 vaccines have been marketed worldwide [15], but only two are licensed in several countries for their use in sows to protect their progeny. Therefore, current products allow applying different vaccination regimes combining piglet and/or sow vaccination [16, 17]. These abovementioned vaccines are an inactivated PCV-2a vaccine, a recombinant vaccine based on an inactivated PCV-1/PCV-2a chimeric virus or subunit vaccines based on a PCV-2a Cap protein. Specifically, the vaccine tested in this study, Ingelvac Circoflex®, is a PCV-2a subunit vaccine based on the product of the ORF2 gene expressed in a baculovirus system [15]. However, cross-protection between the major genotypes worldwide (PCV-2a, PCV-2b and PCV-2d) has been observed in experimental trials and field studies [18–20].

Different studies have shown the capacity of the sow vaccination to induce an immune response and the transfer of passive immunity to the offspring. Sow vaccination before mating stabilizes and homogenizes the PCV-2 immune status of the sow population during gestation [21–24]. Sow vaccination administered before farrowing can confer protection through maternally derived immunity to their offspring against PCV-2 systemic disease by reducing viraemia [16], lesions and viral load in tissues [25] as well as increasing their average daily weight gain in PCV-2 subclinical infection [22]. When this vaccination strategy is boosted in the following reproductive cycles at 3 weeks before farrowing (for 3 years), an improvement of the reproduction rate, number of piglets born alive, birth weight of piglets and number of piglets weaned per a litter was achieved [26]. Another study, where the boost was applied on the second cycle at 2 weeks before farrowing, reported higher number of live-born piglets per litter at the second cycle and higher vitality score from the fist cycle [13] compared to the non-vaccinated (NV) group. Nevertheless, the effect of PCV-2 sow vaccination strategies on reproductive parameters has been, up to now, scarcely studied [13, 26]. Furthermore, practices in the field have explored the option of sow blanket vaccination (personal communication of field veterinarians); however, no contrasted data has been scientifically described.

Hence, the objective of the present work was to evaluate the effects of sow vaccination against PCV-2 applied at different stages of the production cycle (before mating, mid gestation and late gestation), mimicking a blanket vaccination fashion, on productive parameters as well as on virological and serological parameters in sows and their progeny.

## Results

### Productive parameters

Productive parameters from gilts/sows and piglets from the three vaccinated (V) groups taken together and the non-vaccinated (NV) group are shown in Table 1. The comparison of these parameters among each treatment group are detailed in Table 2. In both tables, statistically significant differences and tendencies are indicated.

**Table 1:** Productive parameters (mean ± SD or proportion [percentage] plus confidence interval [CI]) of V and NV sows

	V	NV
**Total born piglets /sow**	14.1 ± 3.0^a^	13.6 ± 3.6^a^
**Live born piglets/sow**	13.4 ± 3.0^a^	12.9 ± 3.7^a^
**Weaning-to-mate interval (days)**	4.6 ± 5.1^a^	4.6 ± 5.0^a^
**Weaning-to-fecundation interval (days)**	30.7 ± 7.0^a^	30.2 ± 5.3^a^
**Weaned piglets/sow**	12.1 ± 2.7^a^	11.8 ± 3.7^a^
**Abortions**	4/180 (2.2%)^a^ CI: 0.1–4.4%	2/53 (3.8%)^a^ CI: − 1.4–8,9%
**Mummies**	34/2424 (1.4%)^a^ CI: 0.9–1,9%	15/695 (2.2%)^a^ CI: 1.1–3.2%
**Stillbirth**	105/2424 (4.3%)^a^ CI: 3.5–5.1%	24/695 (3.5%)^a^ CI: 2.1–4.8%
**Dead suckling piglets**	213/2295 (9.3%)^a^ CI: 8.1–10.5%	58/658 (8.8%)^a^ CI: 6.6–11.0%
**Cross-fostered piglets**	**127/2079 (6.1%)**^**a**^ **CI: 5.1–7.1%**	**53/600 (8.8%)**^**b**^ **CI: 6.6–11.1%**
**Piglets birth weight (Kg)**	**1.64 ± 0.39**^**a**^	**1.58 ± 0.38**^**b**^
**Weaned piglet weight (Kg)**	**6.51 ± 1.48**^**a**^	**6.37 ± 1.48**^**b**^

Different letters in superscript in a row indicate significant differences (*p* ≤ 0.05) between V and NV groups (highlighted in bold)

*V* Vaccinated, *NV* Non-vaccinated

**Table 2:** Productive parameters (mean ± SD or proportion [percentage] plus CI) of the four studied experimental groups

	V PM	V MG	V LG	NV
**Total born piglets /sow**	14.1 ± 2.8^a^	13.9 ± 2.5^a^	14.3 ± 3.5^a^	13.6 ± 3.6^a^
**Live born piglets/sow**	13.3 ± 2.7^a^	13.5 ± 2.6^a^	13.4 ± 3.6^a^	12.9 ± 3.7^a^
**Weaning-to-mate interval (days)**	4.2 ± 3.6^a^	4.7 ± 5.9^a^	4.8 ± 5.7^a^	4.6 ± 5.0^a^
**Weaning-to-fecundation interval (days)**	30.7 ± 7.7^a^	30.6 ± 6.7^a^	30.7 ± 6.6^a^	30.2 ± 5.3^a^
**Weaned piglets/sow**	12.0 ± 2.5^a^	12.2 ± 2.4^a^	12.1 ± 3.1^a^	11.8 ± 3.7^a^
**Abortions**	1/60 (1.7%)^a^ CI: −1.6-4.9%	3/59 (5.1%)^a^ CI: −0.5-10.7%	0/61 (0.0%)^a^ CI: 0.0%	2/53 (3.8%)^a^ CI: −1.4–8,9%
**Mummies**	**9/815 (1.1%)**^**A**^ **CI: 0.4–1.8%**	**10/753 (1.3%)**^**A,B**^ **CI: 0.5–2.1%**	**15/856 (1.8%)**^**A,B**^ **CI: 0.9–2.6%**	**15/695 (2.2%)**^**B**^ **CI: 1.1–3.2%**
**Stillbirth**	37/815 (4.5%)^a^ CI: 3.1–6.0%	27/753 (3.6%)^a^ CI: 2.3–4.9%	41/856 (4.8%)^a^ CI: 3.4–6.2%	24/695 (3.5%)^a^ CI: 2.1–4.8%
**Dead suckling piglets**	74/769 (9.6%)^a^ CI: 7.5–11.7%	67/727 (9.2%)^a^ CI: 7.1–11.3%	72/799 (9.0%)^a^ CI: 7.0–11.0%	58/658 (8.8%)^a^ CI: 6.6–11.0%
**Cross-fostered piglets**	**40/694 (5.8%)**^**a,A,B**^ **CI: 4.0–7.5%**	**33/659 (5.0%)**^**a, A**^ **CI: 3.3–6.7%**	**54/726 (7.4%)**^**a,b,B**^ **CI: 5.5–9.3%**	**53/600 (8.8%)**^**b,A,B**^ **CI: 6.6–11.1%**
**Piglets birth weight (Kg)**	**1.62 ± 0.40**^**a,b**^	**1.66 ± 0.38**^**a**^	**1.64 ± 0.39**^**a**^	**1.58 ± 0.38**^**b**^
**Weaned piglet weight (Kg)**	6.49 ± 1.50^a^	6.52 ± 1.48^a^	6.52 ± 1.45^a^	6.37 ± 1.48^a^

Different letters in superscript in a row indicate significant differences (*p* ≤ 0.05 for lower case letters) or tendency (*p* ≤ 0.10 for capital letters) among experimental groups (highlighted in bold)

*V PM* Vaccinated pre-mating, *V MG* Vaccinated at mid gestation, *V LG* Vaccinated at late gestation, *NV* Non-vaccinated

### PCV-2 antibody values in serum samples of sows and piglets

#### PCV-2 IgG and IgM ELISA optical density (OD) values of sows

**Fig. 1:**
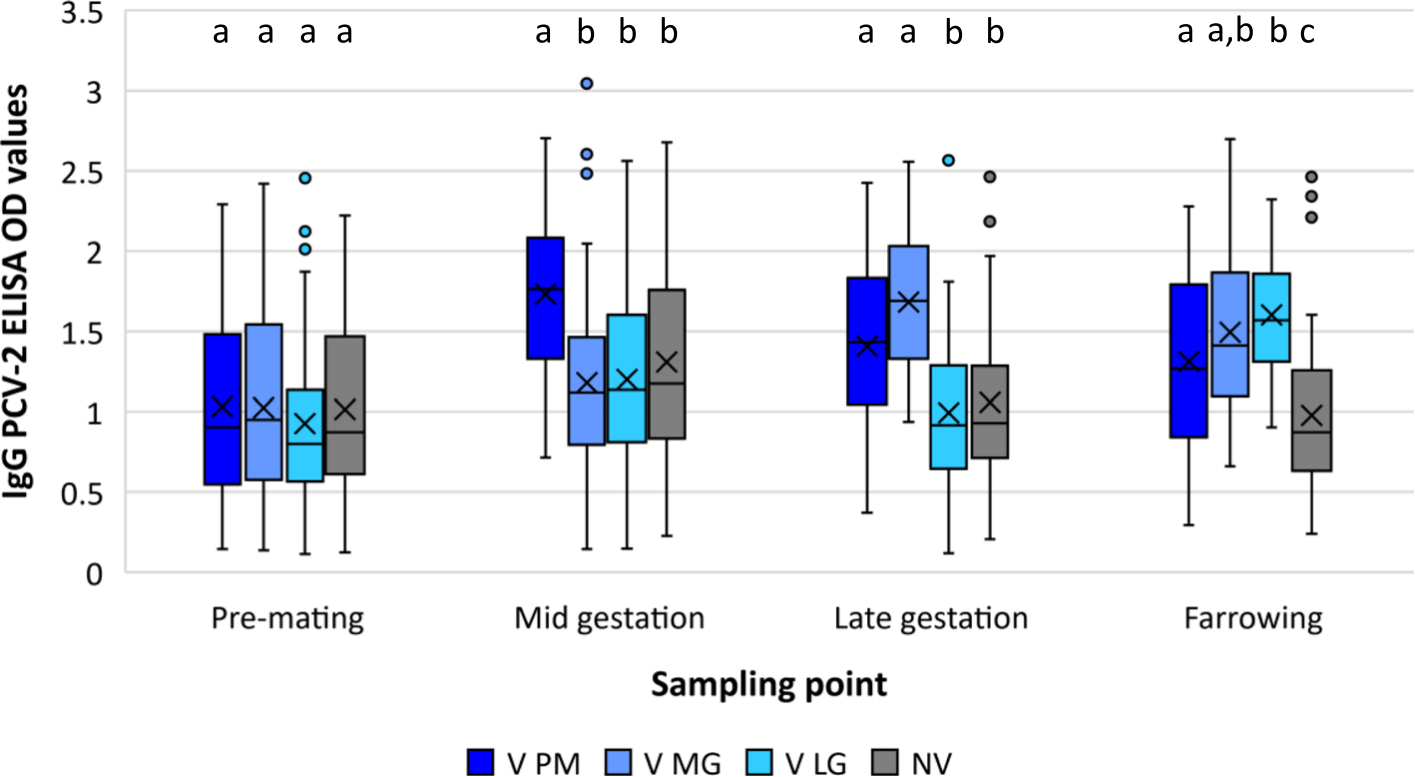
PCV-2 IgG OD results (mean ± SD) form sows’ serum samples of the four experimental groups. V PM: vaccinated pre-mating; V MG: vaccinated at mid gestation; V LG: vaccinated at late gestation; NV: non-vaccinated. Different letters indicate statistically significant differences (*p* ≤ 0.05) among experimental groups for a given sampling point

Sow blood samples collected at the three vaccine/PBS application times as well as at farrowing were used to determine the dynamics of IgG antibodies against PCV-2 (Fig. 1). Each of the different V treatments showed significantly higher IgG values than the NV group from the sampling after their vaccine application until farrowing. At this point, no statistically significant differences between sows vaccinated at mid (V MG) and late (V LG) nor between sows vaccinated at pre-mating (V PM) and V MG group were observed.

In general terms, the PCV-2 IgM OD values of the four groups were very low (mean OD values between 0.40 ± 0.14 and 0.51 ± 0.20 at the different sampling points, data not shown) and no statistical differences in OD values between each experimental group at any time point were found.

#### PCV-2 IgG ELISA S/P values of weaned pigs

**Fig. 2:**
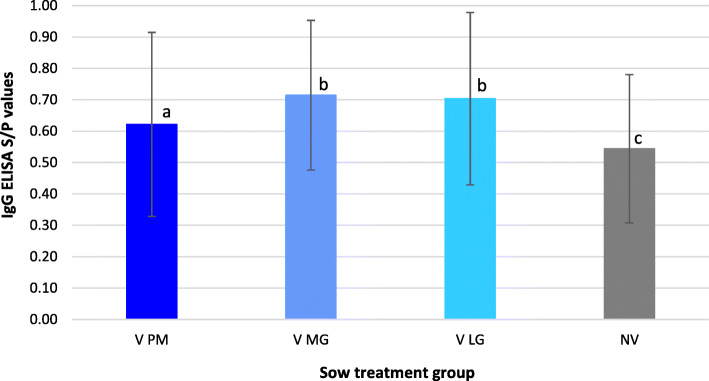
PCV-2 IgG S/*P* values (mean ± SD) from weaned piglets’ serum samples of each experimental group. V PM: vaccinated pre-mating; V MG: vaccinated at mid gestation; V LG: vaccinated at late gestation; NV: non-vaccinated. Different letters indicate significant differences among experimental groups (p ≤ 0.05)

Blood samples from 4 to 6 randomly selected piglets per each sow were taken at weaning and used to detect PCV-2 antibodies. Mean PCV-2 S/P values per treatment groups are represented in Fig. 2. Piglets from V sows showed significantly higher PCV-2 S/P values than piglets from NV sows. The highest values were obtained in piglets from V MG and V LG sows.

### qPCR to detect PCV-2

#### PCV-2 DNA in sow serum samples

Blood samples from sows taken at the three vaccine/PBS application times as well as at farrowing were used to assess the proportion of PCV-2 qPCR positive sera from V and NV animals (Table 3).

**Table 3:** Proportion of PCV-2 qPCR positive sera in sows and CI from V and NV groups

	Sampling point			
	Pre-mating	Mid gestation	Late gestation	Farrowing
**V**	0/186 (0.0%) CI: 0.0%	1/178 (0.6%) CI: −0.5-1.7%	0/177 (0.0%) CI: 0.0%	0/172 (0.0%)^a^ CI: 0.0%
**NV**	0/60 (0.0%) CI: 0.0%	0/52 (0.0%) CI: 0.0%	0/51 (0.0%) CI: 0.0%	2/49 (4.1%)^b^ CI: −1.5-9.6%

Different letters in superscript indicate significant differences (*p* ≤ 0.05) among experimental groups

*V* Vaccinated, *NV* Non-vaccinated

Only three sows were positive to qPCR through the study: One V animal from V PM group was positive at mid gestation sampling point (10^4.2^ PCV-2 copies/mL), and two NV sows were positive at farrowing (one with 10^5.3^ PCV2 copies/mL and another one below the limit of quantification (LOQ = 10^4.00^ PCV-2 genome copies/mL) with 10^3.9^ PCV2 copies/ml). At farrowing sampling point, a lower (*p* = 0.01) proportion of positive PCV-2 qPCR in serum was detected in V sows (0 out of 172) compared to NV ones (2 out of 49).

#### PCV-2 DNA in serum samples from placental umbilical cord pools

Blood from 3 (ranging 1 to 4) porcine umbilical cords (PUCs) per sow were individually collected at farrowing to quantify virus in serum samples. All these samples were processed as a pool of 2–3 PUC sera/sow at farrowing (*n* = 171), except when only one PUC was collected (*n* = 13). The number of PCV-2 qPCR positive pools of PUCs was significantly lower (*p* = 0.01) in V sow groups (12/142 pools, 9% [CI: 3.9–13.0%]) compared to NV group (10/42 pools, 24% [CI: 10.9–36.7%]). When the comparison between each experimental group was performed, animals from V LG group had a significantly lower (*p* = 0.01) proportion of PCV-2 qPCR positive pools of PUCs (2/45 pools, 4% [CI: − 1.6-10.5%]) compared to NV sows (10/42 pools, 24% [CI: 10.9–36.7%]). Additionally, a tendency (*p* = 0.08) in the proportion of PCV-2 qPCR positive pools of PUCs were noted in dams from V MG group (4/47 pools, 9% [CI: 0.5–16.5%]) when compared to NV sows (10/42 pools, 24% [CI: 10.9–36.7%]). Moreover, a lower (*p* = 0.01) PCV-2 load in PUC pools was observed in sows from the V groups (below limit of detection [LOD] [10^0.42^ ± 10^1.43^ PCV-2 genome copies/mL]) compared to NV ones (below LOD [10^1.12^ ± 10^2.11^ PCV-2 genome copies/mL]). No statistically significant differences were observed in PCV-2 load in PUC pools when the comparison was done between each experimental group by separate.

### PCV-2 genotyping

**Fig. 3:**
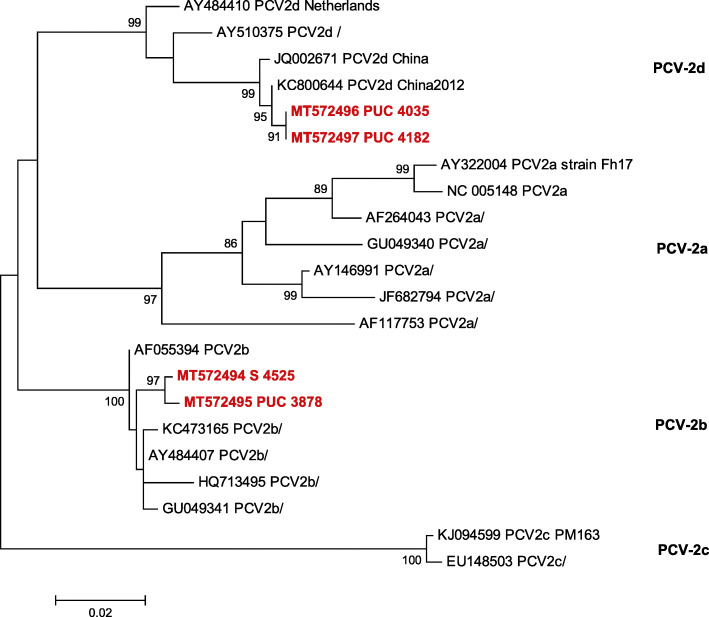
Phylogenetic tree derived from PCV-2 capsid protein (ORF2) sequences. The tree was constructed by using Maximum-Likelihood method with 1000 bootstrap replicates. Bootstrap values higher than 70 are indicated. Sequences from this study are highlighted in red and labelled with the accession number + PUC (for PUC samples) or S (for sow sample) + sow number

PCV-2 ORF2 from serum and PUC samples with some of the highest PCV-2 loads (between 10^4.19^ and 10^8.06^ PCV-2 genome copies/mL) were sequenced to ascertain the main PCV-2 genotype/s circulating in the farm. Specifically, one positive serum sample from the V PM group collected at mid gestation sampling point and 7 individual PUC samples recovered from 3 pools (three from the NV, two from the V MG and two from the V PM sow groups). The phylogenetic tree including the relationships among the ORF2 sequences determined in this study and reference strains is presented in Fig. 3. Genotype PCV-2b was found in the V PM sow serum sample from the mid gestation sampling point (MT572494 S 4525) and in 3 PUC from the same NV sow, all three with identical sequence (MT572495 PUC 3978). These two sequences showed 99,54% of nucleotide identity between them. Genotype PCV-2d was found in two PUC from a V PM sow (both with identical sequence, MT572497 PUC 4182) and in other two PUC from a V MG sow (also with the same sequence, MT572496 PUC 4035). These two sequences were identical between them as well.

## Discussion

The present study deals with a poorly described topic such as the effects of sow vaccination against PCV-2 on productive and infectious parameters of sows and their progeny. To accomplish this task, sows and gilts were vaccinated at three different reproductive periods (before mating, mid gestation and late gestation). When all the vaccinated sow groups were joined (independently of the moment of vaccine application), mimicking a blanket fashion vaccination strategy, significant improvement of productive parameters in terms of piglets’ weight at birth and at weaning and cross-fostering practice reduction were achieved. Moreover, a significant reduction in the proportion of PCV-2 infected sows and their viral loads at farrowing and in PUC were also observed when compared to NV sows, although a low PCV-2 infection pressure was detected. When the period of vaccine application was considered, a tendency in reduction of mummies in the V PM group compared to NV one was observed.

Few published studies describe the benefits of sow vaccination on reproductive parameters under field conditions. This is probably due to the low frequency of reproductive disorders associated with PCV-2 [26], and their rather unknown impact. Currently, only five peer-reviewed published studies recorded reproductive parameters in vaccinated sow farms. In one study [22], dams were vaccinated against PCV-2 at 4 and 2 weeks before artificial insemination and 4 weeks pre-partum. In another one [16], sows were vaccinated 1 week prior to mating. In the third one [26], PCV-2 sow vaccination was implemented for 3 years; dams were vaccinated at 6 and 3 weeks before farrowing in the first reproductive cycle and boosted in the following reproductive cycles at 3 weeks before farrowing. In the fourth study [13], dams were immunized against PCV-2 at 6 and 3 weeks pre-farrowing on the first cycle and sows received a boost 2 weeks pre-farrowing on the second cycle. Finally, in the last one [27], three groups of sows were PCV-2 vaccinated 1 day after weaning, 28 days after weaning and non-vaccinated, respectively. In the present study, in comparison with the abovementioned works, the sow vaccination scheme considered PCV-2 vaccination at different reproductive time points in the same herd, resembling a blanket fashion vaccination strategy.

In the present study, piglets from V sows showed a significantly higher mean birth weight. Among all vaccination groups, the highest body weights were obtained in piglets coming from V MG and V LG groups. Considering the short period between vaccination at late gestation and the delivery, the difference in weight between piglets from V LG and NV groups was unexpected. This result, however, would be similar to the one obtained in one study in which sows were also vaccinated at late gestation [26]. Moreover, the weight of weaned piglets (at 3 weeks of age approximately) coming from V sows was higher compared to those from NV dams. These results are in contrast with the ones of Fraile et al. [16], where sows were vaccinated pre-mating and no differences were observed in weaned piglet weight at 4 weeks of age. Reasons for such differences may be attributable to the different vaccine product or schedule used and/or lack of power due to the limited sample size of the study. Moreover, there are many inter-farm factors (animal genetics, farm facilities, management practices, treatments, nutrition and vaccination schedule) that may influence the vaccination outcome.

Piglets coming from V sows were significantly less cross-fostered compared to the NV group; more specifically, significantly less cross-fostering was performed in V PM and V MG dams compared to the NV group. Also, a tendency to practice less cross-fostering in piglets from V MG dams was noted compared to that in V LG sows. Cross-fostering is a practice frequently used to increase piglet survival and to organize litters with uniform body weight [28]. However, biosecurity procedures recommend minimizing the number of cross-fostered animals [29]; therefore, reduction of this practice may help diminishing PCV-2 transmission among piglets.

A tendency of a lower proportion of mummies was observed in the V PM sow group compared with the NV group. Considering that mummification is an outcome of late reproductive problems [30], the potential benefits of PCV-2 vaccination at pre-mating on mummies reduction was expected. This result is in contrast with Kurmann et al. [22] and Sibila et al. [24], where sows were vaccinated pre-mating, and with Oliver-Ferrando et al. [13], where sows were vaccinated at mid-late gestation. Further studies with a higher number of tested sows would be needed to confirm the data obtained herein.

In relation to stillborn, no statistical differences were observed in the current study, in accordance with Cybulski et al. study [27]. Oliver-Ferrando et al. [13] found an inconsistent situation, where a higher number of stillbirths/litter in the V group was observed in the first reproductive cycle but not in the second one. Nevertheless, proportions of stillborn detected in the present study and in Oliver-Ferrando et al. [13] are aligned with regular values expected in the average Spanish pig farm (8.6%) according to national records (www.bdporc.irta.es).

Although Oliver-Ferrando et al. [13] and Pejsak et al. [26] reported an improvement in number of liveborn piglets, in the present case only a positive (numerically, but non-significant) effect was obtained similar to the study of Kurmann et al. [22] and in 28 days after weaning vaccination group from Cybulski et al. study [27].

In addition, no significant differences in the proportion of abortions were detected in the present study similarly to results obtained in Kurmann et al. study [22]. Considering the low frequency of abortions detected in the present study, a larger sample size would be necessary to analyse this parameter and determine their effect. Nevertheless, the design of our study was not within the scope of detecting the presence of significant differences for this latter parameter.

Similarly, Pejsak et al. [26] and Oliver-Ferrando et al. [13] detected a higher number of weaned piglets/litter (being only statistically significant in Pejsak) in V dams compared to NV dams, respectively. These two latter studies indicated that the repeated use of PCV-2 sow vaccination can improve reproductive parameters. So, these positive effects (piglets born alive and weaned piglets/litter) may probably be more evident after several gestational cycles vaccinating the sows against PCV-2 rather than one single vaccination of sows as in the present study.

The antibody profile from sows immunized against PCV-2 revealed seroconversion after each vaccination, conferring a stronger herd immunity against PCV-2 compared to the NV [10, 13, 22, 24]. Besides, the NV group maintained low ELISA OD values during all gestation period. In this sense, despite sow infection remained low or non-detectable in all studied groups during the gestation period, no V sows were found infected at farrowing while 4% of NV ones were qPCR positive. Although significant differences at the farrowing period in terms of percentage of viraemic sows was detected, the number of PCV-2 qPCR positive sera in sows was low. These results, however, should not be considered surprising since the number of infected sows tend to be low in most farms where subclinical infection is taking place [8, 13, 31, 32]; moreover, batch differences may also account for certain variability. Therefore, further studies would be desirable to further confirm this finding.

The virological results obtained in the study were in fairly contrast with those obtained in the screening prior to the start of the study, where PCV-2 DNA was detected in serum samples from 18 out of 30 clinically healthy sows. Moreover, PCV-2 genome was also detected in 24 out of 30 PUCs from the same sows. These initial results would fit well with those observed by other research groups in some PCV-2-SI farms in absence of vaccination [33]. It is very likely that differences on percentage of infected sows among batches and the PCV-2 vaccination in ¾ of the studied groups could explain the variability and reduction of the PCV-2 infection pressure within the same farm when the large study was carried out. It is speculated that the reduction of the PCV-2 infection pressure probably caused a reduction of transplacental transmission of PCV-2 to foetuses, evidenced by the lower proportion of PUC PCV-2 qPCR positive samples and viral load detected compared to the farm previous screening.

The higher antibody levels of V sows than NV ones at farrowing suggests a higher transfer of maternally derived antibodies (MDA) to piglets of V sows via colostrum as also described [16, 22, 34]. This scenario should place piglets from V sows in a better immune position to counteract PCV-2 infection at early ages [16]. As expected, the highest MDA transfer was observed in weaned piglets from V MG and V LG groups, followed by V PM. On the other hand, considering that all piglets were vaccinated at 3 weeks of age against PCV-2, certain concerns regarding MDA interference with vaccination could arise. Although in this study MDA interference with pig vaccination was not explored, several studies have reported evidence of such interference with vaccine humoral immune response [16, 35–38]. However, the efficacy of the same vaccine used in this study has not shown to be jeopardized by high values of MDA [39].

Two PCV-2 genotypes, PCV-2b and PCV-2d, circulated in the herd, being the most prevalent ones in the field worldwide [40, 41]. The co-circulation of two or more PCV-2 genotypes in the same farm is not unusual [42–44]. Current vaccines based on PCV-2a strains appear to be able to cope with major circulating strains worldwide due to cross-protection among genotypes [3, 18–20].

PCV2 vaccines are the most sold preventive product in the porcine industry and one of the biological products in swine with highest return of inversion [15]. Besides, the combined vaccination of sows and piglets is an increasing practice since provides the optimal performance in animals [16, 45], and although there is little information about gilt/sow immunization, vaccination of pigs and sows has been estimated cost-efficient [15]. However, each farm should be analysed case by case to study the cost /benefit of PCV-2 vaccination [46].

## Conclusions

Vaccination of the breeding herd (gilts and sows) against PCV-2, mimicking a blanket fashion schedule in a subclinical infection scenario, improved immune status of dams and the progeny against the virus and reduced virus circulation at farrowing in sows and vertical infection to foetuses, although a low infectious pressure was detected during the study. PCV-2 sow vaccination also improved piglets’ weight at birth and at weaning and reduced cross-fostering practice. Also, a tendency in reduction of mummies from V PM group compared to NV was observed. Due to the limited PCV-2 circulation detected in the dams during the present exploratory study, the impact of PCV-2 vaccination on the productivity was considered of low impact. Further studies should be needed to confirm the currently obtained results.

## Materials and methods

### Farm selection

Inclusion criteria for farm selection were: a) sows/gilts housed in a single conventional farm (site I), b) no PCV-2 vaccination schedule in gilts/sows, c) allowance of controlled cross-fostering practices, and d) evidence of PCV-2 infection by means of viral genome detection in sows and PUCs in site I. Indeed, prior to the start of the study, PCV-2 DNA was detected in serum samples from 18 out of 30 (60%, viral load range 10^3.50^ and 10^7.91^ genome copies/mL) clinically healthy sows from different parity number and in 24 out of 30 (80%, viral load range 10^3.50^ and 10^7.06.^ genome copies/ml) PUCs from the same sows, indicating a PCV-2-SI scenario. Inclusion criteria for gilts/sows selection were: a) healthy animals, b) not pregnant, c) from the same genetic line, d) of all parities (gilts, primiparous and multiparous).

The study was conducted in a two-site commercial farm located in Catalonia (Spain). The farm had 1400 sows (including gilts) and used a weekly farrowing batch system; piglet weaning was performed at 25 days of age approximately. The farm was *Mycoplasma hyopneumoniae* positive, *Porcine reproductive and respiratory syndrome virus* (PRRSV) positive stable, and negative for Aujeszky’s disease virus. Gilts and sows were crossbred (Duroc x Landrace) and were artificially inseminated with Pietrain boar semen. The vaccination routine of the farm included gilt and sow immunization against PRRSV, *Porcine parvovirus, Erysipelothrix rhusiopathiae, Escherichia coli* and *Clostridium perfringens*. Piglets were vaccinated against PCV-2 and *Mycoplasma hyopneumoniae* before weaning.

### Study design

**Fig. 4:**
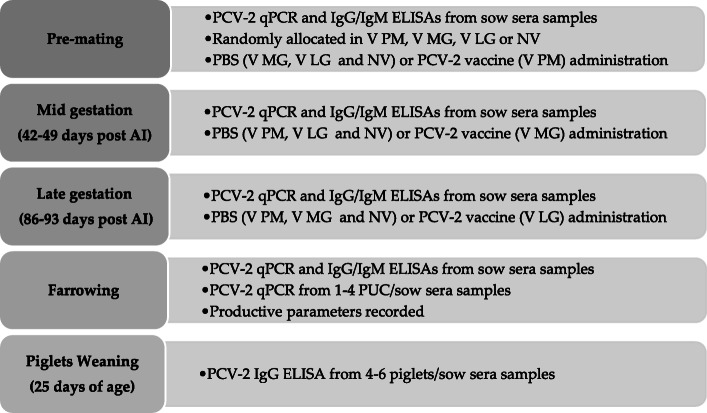
Experimental study design, including sampling time points and PCV-2 vaccine/PBS application timing. V PM: vaccinated at pre-mating; V MG: vaccinated at mid gestation; V LG: vaccinated at late gestation; NV: non-vaccinated; AI: artificial insemination; PCV-2 vaccine: 1 mL of PCV-2 Ingelvac Circoflex®; PBS: phosphate buffer saline

The present study was a parallel group, randomised and controlled trial. The study was unmasked for personnel involved in study design and monitoring, vaccine dispensation and for personnel involved in body weight and sample collection, whereas it was masked for farm personnel (routine management, daily observation and routine reproductive data recording) as well as for laboratory technicians. Study design is summarized in Fig. 4. Sows were bled before mating and tested for PCV-2 IgG and IgM ELISAs. Animals were randomly allocated into 4 different treatment groups blocking by parity (from 0 to 8) and OD values for PCV-2 IgG and PCV-2 IgM. A total of 288 healthy sows were selected through six consecutive breeding batches and distributed in the following treatments groups: vaccination of 73 at pre-mating (PM), 72 at mid gestation (MG), 73 at late gestation (LG) and 70 were kept as NV sows. These three specific immunization times were chosen to mimic a blanket vaccination fashion, which is performed in sows at all physiological status at a given time point. Gestation was monitored until farrowing and then their piglets were followed up until weaning. Sows from all treatment groups were housed comingled during the study (in same pens when they were housed in groups, and in same room when they were individualized). Cross-fostering of piglets was only allowed among sows of the same experimental group.

All sows included in the study were injected (with phosphate buffered saline [PBS] or PCV-2 vaccine) three times before farrowing. Sows were vaccinated intramuscularly (neck muscle) with 1 mL of PCV-2 Ingelvac Circoflex® or PBS. Blood samples from sows were collected at the three vaccine/PBS application times as well as at farrowing. Obtained sera were tested for PCV-2 IgG and IgM antibodies using a commercial ELISA assay and to quantify virus using a qPCR assay. At pre-mating sampling point, PCV-2 IgG and IgM ELISA assay was performed in 288 sow sera to randomize the sows in 4 experimental groups. ELISA assay from mid gestation, late gestation and farrowing sampling points and qPCR assay from the fourth sampling points were performed only in those artificially inseminated sows (Additional file 1). Blood from 3 (ranging 1 to 4) PUCs per sow were individually collected at farrowing to quantify virus in serum samples using the qPCR assay. In order to minimize PCV-2 environmental contamination, gloves were changed, and scissors were disinfected with ethanol for each PUC sampling. Additionally, productive parameters from gilts/sows and piglets were recorded (described in section 7). Moreover, at weaning, sera samples from 4 to 6 randomly selected piglets per each sow were taken and used to detect PCV-2 antibodies using an ELISA test.

Once in the laboratory, blood samples were centrifuged at 2500 rpm (1300 g) during 10 min at 4 °C to obtain sera. All sera were stored at − 20 °C until testing.

### DNA extraction and qPCR

Presence of PCV-2 DNA by qPCR was assessed in the serum samples from sows and PUCs. All these samples were processed by pools. Indeed, pools from 2 to 3 sow serum samples at each sampling point and pools of 2–3 PUC sera per sow at farrowing (except when only one PUC was collected) were created. When a pool from sow sera was qPCR positive, individual serum samples were tested by qPCR following the same protocol.

DNA was extracted from 200 μL of serum (from sows) or pool by using the MagMAX™ Pathogen RNA/DNA Kit (Thermo Fischer Scientific Baltics. Vilnius, Lithuania) following the manufacturer’s instructions. To quantify the PCV-2 DNA in serum samples, a qPCR assay (LSI VetMAX™ Porcine Circovirus Type 2-Quantification, Applied Biosystems, Lisseu, France) was performed. Each extraction and qPCR plate included negative controls (diethylpyrocarbonate [DEPC]-treated water) and each sample reaction had an internal positive control to monitor DNA extraction and amplification procedures.

PCV-2 qPCR results were transformed as follows:

- Undetermined results and those below LOD (LOD = 10^3.50^ PCV-2 genome copies/mL) were transformed as log_10_ (0 + 1).
- Results between LOD and LOQ (LOQ = 10^4.00^ PCV-2 genome copies/mL) were transformed following the method proposed by Croghan et al. [47], where the result was calculated as log_10_ ((LOQ/√2) + 1). Therefore, the imputed value was log_10_ (7071 + 1).
- Results over LOQ were transformed as log_10_ (PCV-2 genome copies/mL + 1).

### PCV-2 antibody detection by ELISA

PCV-2 antibodies in sows were detected at the three vaccine/PBS application times as well as at farrowing using the ELISA kit Ingezim Circo IgG/IgM 11.PCV.K2® assay (Ingenasa, Madrid, Spain). ELISA results were expressed as mean OD (± SD) according to the kit instructions.

PCV-2 antibodies in piglets at weaning were detected using the ELISA kit Ingezim Circo IgG 11.PCV.K1® assay (Ingenasa, Madrid, Spain). Mean positive cut-off was established as OD of negative control + 0.25). ELISA results from piglets were expressed as S/P ratio (OD of sample / OD of positive control for each ELISA plate) following the manufacturer’s recommendations.

### PCV-2 ORF2 amplification and sequencing

Capsid protein gene (ORF2) was sequenced from PCV-2 qPCR positive sow serum and PUC samples to determine the PCV-2 genotype/s circulating in the farm. DNA was extracted from serum samples by using the MagMax™ Pathogen RNA/DNA Kit (Thermo Fischer Scientific Baltics. Vilnius, Lithuania) following the manufacturer’s instructions.

PCV-2 Cap gene was amplified from nucleotide 1050 to 1735 (PCV-2 genome; GenBank Accession Number: AY181948) using the primers PCV-2all_F (5′ GGGTCTTTAAGATTAAATYC 3′) and PCV-2all_R (5′ ATGACGTATCCAAGGAG 3′) [48]. PCR was performed in a 25 μL reaction containing 5 μL of PCR Promega buffer, 2.5 μL of 25 Mm MgCl_2_, 1.25 μL of each primer at 10 pmol/μL, 1 μL of 5 mM dNTPs, 0.15 U of Taq DNA polymerase, 11.35 μL of DEPC-treated water and 2.5 μL of extracted DNA. The PCR was performed with the following program: denaturation of 5 min at 94 °C, 40 cycles of 30 s at 95 °C for denaturation, 30 s at 53 °C for primer annealing and 40 s at 72 °C for elongation, with a final elongation of 7 min at 72 °C. Amplified DNA was confirmed by electrophoresis gel with 2% agarose.

NucleoSpin Gel and PCR Clean-up kit (Macherey–Nagel, GmbH & Co. KG, Dueren, Germany) was used to purify the PCR product. BigDye Terminator v3.1 Cycle Sequencing Kit (Applied Biosystems, Foster City, CA, USA) and 3130 × l Genetic Analyser (Applied Biosystems, Ohio, USA) was used to perform the sequencing reaction and the analysis, respectively [48]. Sequences were edited and assembled by using ChromasPro Version 2.1.8 (Technelysium). The sequences obtained were submitted to the GenBank with the following accession numbers MT572494-MT572497).

### PCV-2 capsid ProteinG (ORF2) phylogenetic and sequence analysis

To genotype the PCV-2 sequences obtained, an alignment with 18 representative sequences from genotypes PCV-2a, b, c and d was carried out with Clustal Omega (EMBL-EBI). The phylogenetic tree was created by using the Maximum Likelihood method included in Mega-X software [49]. The best substitution model according with the Bayesian information criterion was the Hasegawa-Kishino-Yano model, with a discrete Gamma distribution. Bootstrap resampling test was carried out with 1000 replicates. Bootstrap values higher than 70 were indicated in the constructed phylogenetic tree.

### Statistical analyses

Statistical analyses were carried out using the software NCSS (Kaysville, Utah, USA). Comparisons were performed in two ways: 1) all V groups (mimicking a blanket fashion PCV-2 vaccination) versus the NV group, and 2) all experimental groups among them.

When comparison was performed between V and NV groups Mann-Whitney U test was used to total born piglets per sow, live born piglets per sow, weaned piglets per sow, weaning-to-mate interval, weaning-to-fecundation interval, PCV-2 viral load in sera of sows as well as viral load in PUC. Chi-square test was used to compare proportions of abortions, mummified fetuses, stillbirth piglets, dead suckling piglets, cross-fostered piglets, as well as of viraemic sows and PUC. The T –test was used to analyse birth and weaning body weights.

When comparison was performed among all experimental groups, Kruskal Wallis test (including Bonferroni test for multiple comparison) was used to analyse total born piglets per sow, live born piglets per sow, weaned piglets per sow, weaning-to-mate interval, weaning-to-fecundation interval, PCV-2 ELISA antibody values in sow and piglets’ serum samples and viral load in sera of sows and PUCs. Serological parameters from sows were analysed performing only the comparison among experimental groups to assess the serological effect of each vaccination. Chi-square or Fisher’s Extact test was used to compare proportion of abortions, mummified piglets, stillbirth piglets, dead suckling piglets, cross-fostered piglets, as well as of viraemic sows and PUCs. Moreover, ANOVA (including Tukey-Kramer multiple comparison test) was used to compare birth and weaning body weights.

The significance level (α) was set at *p* ≤ 0.05, whereas statistical tendencies were reported when *p* ≤ 0.10.

## Supplementary Information

**Additional file 1.** Number of animals analysed at each sampling point.

## Data Availability

The datasets used and/or analysed during the current study are available from the corresponding author on reasonable request.

## Abbreviations

DEPC: Diethylpyrocarbonate
ELISA: Enzyme-linked immunosorbent assay
LOD: Limit of detection
LOQ: Limit of quantification
MDA: Maternally derived antibodies
NV: Non-vaccinated
OD: Optical density
PBS: Phosphate buffered saline
PCV-2: *Porcine circovirus 2*
PRRSV: *Porcine reproductive and respiratory syndrome virus*
PUC: Placental umbilical cord
qPCR: Real time quantitative polymerase chain reaction
SD: Standard Deviation
V LG: Vaccinated at late gestation
V MG: Vaccinated at mid gestation
V PM: Vaccination at pre-mating
