# Supplementary material for: Exploratory field study on the effects of porcine circovirus 2 (PCV-2) sow vaccination at different physiological stages mimicking blanket vaccination
Source: Porcine Health Manag. 2021 Apr 26;7:35. doi: 10.1186/s40813-021-00213-2 (PMC8077688; doi:10.1186/s40813-021-00213-2)
Supplement: Supplementary file 1 — Additional file 1. Number of animals analysed at each sampling point. [file 40813_2021_213_MOESM1_ESM.docx]

**Additional file 1:** Number of animals analysed at each sampling point.

| Group | Sows at: | | | | | | | | | Piglets at weaning |
| --- | --- | --- | --- | --- | --- | --- | --- | --- | --- | --- |
|  | **Pre-mating*** | | **Mid gestation** | | **Late gestation** | | **Farrowing** | | |  |
|  | **ELISA** | **qPCR** | **ELISA** | **qPCR** | **ELISA** | **qPCR** | **ELISA** | **qPCR** | **qPCR PUC Pools**** | **ELISA***** |
| V PM | 73 | 63 | 60 | 60 | 60 | 60 | 58 | 58 | 50 | 294 |
| V MG | 72 | 61 | 57 | 57 | 56 | 56 | 54 | 54 | 47 | 272 |
| V LG | 73 | 62 | 61 | 61 | 61 | 61 | 60 | 60 | 45 | 300 |
| NV | 70 | 60 | 52 | 52 | 51 | 51 | 49 | 49 | 42 | 139 |
| Total | **288** | **246** | **230** | **230** | **228** | **228** | **221** | **221** | **184** | **1005** |

*The number of samples tested by ELISA referred to the animals sampled to be distributed within the groups (pre-screening); the qPCR results referred to those sows with confirmed pregnancy. The number of samples tested by ELISA and qPCR at the remaining sampling points referred to the number of sows available at each time point.

**Pools with 2-3 PUC per sow were constructed. Two sows with 4 PUC were analysed in 2 pools of 2 PUC each pool.

*** Sera samples from 4 to 6 randomly selected piglets per each sow were taken and used to detect PCV-2 antibodies using an ELISA test.
